# *Lycopus lucidus* Turcz Inhibits the Osteoclastogenesis in RAW 264.7 Cells and Bone Loss in Ovariectomized Rat Model

**DOI:** 10.1155/2019/3231784

**Published:** 2019-02-21

**Authors:** Da-Won Jeong, Eun-Young Kim, Jae-Hyun Kim, Bina Lee, SooYeon Hong, Jae Ho Park, Hyuk-Sang Jung, Youngjoo Sohn

**Affiliations:** ^1^Department of Anatomy, College of Korean Medicine, Kyung Hee University, Seoul 02447, Republic of Korea; ^2^Department of Medicinal Plant Science, Jungwon University, 85 Munmu-ro, Goesan-eup, Goesan-gun, Chungbuk, Republic of Korea

## Abstract

*Lycopus lucidus* (LL) is a perennial herb that is traditionally used in Asia to treat edema, wound healing, and gynecological diseases such as irregular menstruation and menstrual pain. We hypothesized that LL would decrease the risk of developing osteoporosis, which is a condition related to gynecological diseases. In this study, we aimed to investigate the effect of a water extract of LL on osteoclastogenesis* in vitro* and osteoporosis* in vivo*.* In vitro* study, we used RAW 264.7 cells as osteoclast precursor cell. Osteoclast differentiation was induced by receptor activator nuclear factor-kappa B ligand (RANKL). We investigated the effect of LL on RANKL-induced osteoclastogenesis, tartrate-resistant acid phosphatase (TRAP) activity, and osteoclast-related genes.* In vivo* study, we used ovariectomized- (OVX-) induced osteoporosis rat model. OVX-induced Sprague-Dawley rats were randomly separated into sham, OVX, 17*β*-estradiol (100 *μ*g/kg), wLL-L (15.2 mg/kg), and wLL-H (152 mg/kg) groups. Drugs were administered orally once daily for 9 weeks. wLL inhibited the formation of TRAP-positive osteoclasts; TRAP activity; pit formation; transcription factors (the nuclear factor of activated T-cell cytoplasmic 1 and c-fos); and osteoclast-related genes such as TRAP, carbonic anhydrase II, cathepsin K, osteoclast-associated receptor, and the d2 isoform of the vacuolar ATPase Vo domain. Also, wLL prevented loss of the trabecular area in the OVX femur without change of estrogen level. These results indicate that wLL is able to inhibit osteoclastogenesis and protect bone loss in the OVX-induced osteoporosis model without the influence of hormones like estrogen.

## 1. Introduction

Osteoporosis is a systemic skeletal disease characterized by a loss of bone mass and deterioration of the bone microarchitecture; it results in fractures caused by bone weakening [1]. Bone mass, on the other hand, is maintained by the turnover of bone resorption by osteoclasts and bone formation by osteoblasts [2]. Aging, menopause, and the abuse of steroids such as glucocorticoid cause osteoporosis by inhibiting sufficient bone turnover [1, 3].

Osteoclasts are multinucleated cells formed by the fusion and differentiation of mononucleated hematopoietic cells; they play a major role in bone turnover and pathological conditions like osteoporosis [4]. Excessive osteolysis of osteoclasts leads to lytic bone diseases, such as osteoporosis, rheumatoid arthritis, Paget's disease, and periodontitis [5, 6]. Therefore, studying the inhibition of osteoclast activity and proliferation is useful for developing new drug to treat osteolytic disease. The differentiation and activation of osteoclasts cannot take place without the receptor activator nuclear factor-kappa B ligand (RANKL), which is produced by osteoblasts [7]. The interaction of RANKL and its receptor, RANK, induces the expression of the nuclear factor of activated T cells (NFATc1) through tumor necrosis factor receptor-associated factor 6 (TRAF6) and c-fos [8]. NFATc1, as a master regulator of osteoclastogenesis, induces the tartrate- resistant acid phosphatase (TRAP), carbonic anhydrase II (CA II), cathepsin K (CTK), and osteoclast-associated receptor (OSCAR) concerned with osteoclastogenesis and the d2 isoform of vacuolar ATPase Vo domain (ATP6v0d2) relative with cell fusion [9, 10].

*Lycopus lucidus* Turcz (LL) is a plant that belongs to the Lamiaceae family. In Asia, its leaves and stems are harvested before the plant blossom. These are then dried and have traditionally been used to treat edema, wound healing, pain, and gynecological disease such as irregular menstruation and menstrual pain [11]. According to recent research, LL consists of rich flavonoids, coumarins, terpenoids, and tannins and has antiallergic [12], anticancer [13], and antioxidant effects [14]. Pankova and Tsvetkova reported that flavonoids enhanced bone formation and inhibit bone resorption [15], while Tang et al. showed that coumarin derivatives stimulate bone formation in osteoporosis diseases [16]. Therefore, we expect that LL will inhibit bone resorption by osteoclasts and bone loss in osteoporosis disease. In this study, we have investigated the effect of LL on both Raw 264.7 cells in vitro and in an OVX-induced osteoporosis model in vivo. The results of this study can serve as a basis for the development of drugs to treat osteoporosis.

## 2. Method and Materials

### 2.1. Reagents

Dulbecco's modified eagle's medium (DMEM) was purchased from Welgene (Daejeon, Korea). An alpha-minimal essential medium (*α*-MEM), penicillin-streptomycin solution, and fetal bovine serum (FBS) were purchased from Gibco (Grand Island, NY); recombinant mouse RANKL was purchased from Peprotehc (London, UK); an osteo assay surface plate was obtained from Corning, Inc. (Corning, NY); a cell titer 96 aqueous nonradioactive cell proliferation assay kit was obtained from Promega (Madison, WI); antiactin and anti-c-fos were obtained from Santa Cruz Biotechnology (Santa Cruz, CA); anti-NFATc1 was purchased from BD pharmingen (San Diego, CA); a reverse-transcription kit was purchased from Invitrogen (Carlsbad, CA); and the taq polymerase was purchased from Kapa Biosystem (Wilmington, MA). All primer pairs were made by Genotech (Daejeon, Korea). In addition, the TRAP staining kit, 17b-estradiol (E2), and kaempferol were purchased from Sigma-Aldrich (St. Louis, MO); isoflurane was purchased from Hana Pharm (Hwasung, Korea); and pentobarbital sodium was obtained from Hanlim Pharm (YoungIn, Korea).

### 2.2. Preparation of LL and High Performance Liquid Chromatography (HPLC) Analysis

LL was purchased from the Kyung Hee University Medical Center (Seoul, Korea). The LL voucher specimen (A065) was deposited in Department of anatomy, College of Korean Medicine, Kyung Hee University. The dried LL (500g) was decocted with 5L of boiling distilled water (DW) for 2 hours and then cooled at room temperature. The extract was filtered using filter paper (Whatman no. 3, Maidstone, UK), concentrated with a vacuum evaporator, and then lyophilized. The yield of LL was 11.4%.

The HPLC analysis was carried out on the Waters 2695 system with a 2996-dual *λ* absorbance detector. The column was equipped with the XBridge™ C18 column (250 mm × 4.6 mm 5 *μ*m). The mobile phase consisted of acetonitrile (solvent A) and 1% acetic acid (solvent B) at a flow rate of 1.0 mL/min. The injection volume of the extract was 10 *μ*L. The elution phase was as follows: 0-40 min of 10-40% solvent A and 90-60% solvent B. The elution was monitored at 368 nm.

### 2.3. Cell Culture and Viability

RAW 264.7 cells were purchased from a Korean cell line bank (Seoul, Korea) and grown in DMEM supplemented with 10% FBS and penicillin (100 units/mL) and streptomycin (100 *μ*g/mL) in a humidified atmosphere of 5% CO2 at 37°C. To measure cell viability, the cells were seeded at 5 × 10^3^ cells per well in a 96-well plate. After 24 hours, the medium was changed and treated with the LL of various concentrations (1, 10, and 100 *μ*g/mL) for 24 hours. Then 20 *μ*L of the MTS reagent was added the well plate and incubated at 37°C for 2 hours. The optical density was measured at 490 nm with an enzyme-linked immunosorbent assay plate reader.

### 2.4. TRAP Staining and Pit Formation

To induce osteoclastogenesis, the RAW 264.7 cells were seeded on a 96-well plate or osteo assay surface plate at a density of 5 × 10^3^ cells per well in *α*-MEM medium. After 24 hours, the cells were incubated with *α*-MEM supplemented with RANKL (100 ng/mL) and LL of various concentrations (1, 10, and 100 *μ*g/mL) for 5 days. The culture medium was replaced every 2 days. For the osteoclast staining, cells were washed with DPBS and fixed with 4% formalin. The cells were then stained with an acid phosphatase leukocyte kit following the manufacturer's instructions. TRAP-positive cells with more than 3 nuclei were counted as osteoclasts. TRAP activity was measured with the cell culture medium using an acid phosphatase leukocyte kit. To measure the rate of bone resorption, the plate was incubated with 4% sodium hypochlorite for 30min to remove the cells. The pit area was measured using Image J.

### 2.5. Western Blot Analysis

RAW 264.7 cells were seeded in a 60mm cell culture dish at the density of 5 × 10^5^ cells per well in *α*-MEM medium. After 24 hours, the cells were incubated with *α*-MEM supplemented with RANKL (100 ng/mL) and LL of various concentration (1, 10, and 100 *μ*g/mL) for 24 hours. The cells were lysed in RIPA buffer. Equal amounts of proteins were separated by SDS-PAGE and transferred using an electro transfer kit to nitrocellulose membranes (Whatman, UK). The membranes were blocked with 5% skim milk; probed for 24 hours with the specific primary antibodies such as c-fos and NFATc1, at 4°C; and then incubated with horseradish peroxidase conjugated secondary antibodies for 1 hour. Immunoreactivities were detected using an Amersham™ ECL™ Western blotting detection reagent (Buckinghamshire, UK).

### 2.6. Reverse-Transcription PCR

RAW 264.7 cells were incubated with *α*-MEM supplemented with RANKL (100 ng/mL) and LL of various concentrations (1, 10, and 100 *μ*g/mL for 4 days. Total RNA was extracted using a TriZol reagent (TaKaRa Bio, Ostu, Japan). Two micrograms of RNA were converted to complementary DNA (cDNA) using superscript II reverse-transcriptase (Invitrogen, Carlsbad, CA, USA). Each cDNA was amplified using specific primers. The specific primer sequence and reaction condition are summarized in Table 1.

### 2.7. Animals and the Ethical Use of Animals

Forty female Sprague-Dawley rats (12 weeks old) were purchased from Nara Biotech (Seoul, Republic of Korea). The rats were kept in standard conditions (22°C ± 2°C, 50% ± 5% humidity, 12-hour light/dark cycle). Prior to the experiment, all animals were adapted to the experimental environment for 1 week. All rats were provided a commercial Purina rat and mouse diet (Purina chow no. 38057; Cargill Agri Purina Inc., Seoul, Korea). All animal experiments were performed in accordance with the University's Guidelines for the Care and Use of Laboratory Animals approved by the Committee on Animal Experimentation of Kyung Hee University (permission number: KHUASP(SE)-15-101).

### 2.8. OVX Surgery and Study Design

Rats were deeply anesthetized using 5% isoflurane with NO_2_/O_2_ (7:3) prior to the surgery, and anesthesia (2.5% isoflurane with NO_2_/O_2_ [7:3]) was maintained during the surgery. Thirty-two rats were bilateral ovariectomized (OVX), and eight rats underwent a sham operation. The OVX rats were randomly divided four groups of eight rats each: an OVX group, an E2 group (17*β*-estradiol, 100 *μ*g/kg), a wLL-L group (wLL-low dose-treated group, 15.2 mg/kg), and a wLL-H group (wLL-high dose-treated group, 152 mg/kg). The sham and OVX groups were orally administered DW (1mL) instead of drug for 8 weeks. The E2, wLL-L, and wLL-H groups were orally administered each drugs dissolved in the DW of the same volume. The body weight of each rat was measured once a week for 8 weeks. E2 was served as a positive control. LL dose was calculated as follows. A single dose for LL is 8g per 60kg per day, that is equal to 0.91g of the LL extract per 60kg adult. In this study, the wLL-L group was orally administered a weight ratio (15.2 mg/kg). wLL-H group was orally administered 10 times higher than wLL-L (152 mg/kg)

### 2.9. Bone Histological and Histomorphometric Analysis

After 8 weeks of treatment, the rats were sacrificed under anesthesia with pentobarbital sodium (80 mg/kg, i.p), and their femur, liver, and uterus were removed immediately and weighed on an electronic scale. The femurs were fixed with neutral buffered formalin, washed with running water for 24 hours, and then were decalcified with 10% EDTA-2Na for 3 weeks. The decalcified femurs were washed with running water, dehydrated, and embedded with paraffin. The embedded blocks were sliced using a Leica RM 2125 RTS microtome (Leica, Bensheim, Germany) into 5 *μ*m sections, which were then stained with hematoxylin and eosin to measure the trabecular area. The other sections were stained with TRAP using a TRAP staining kit to visualize the osteoclasts.

### 2.10. Statistical Analysis

All experiments were repeated at least three times and the data are presented as mean ± standard error. The statistical analysis was performed using GraphPad Prism 5.01. Raw data were subjected to one-way ANOVA followed by Dunnett's post hoc comparisons. A* p-*value less than 0.05 was considered statistically significant.

## 3. Results

### 3.1. HPLC Analysis

**Figure 1:**
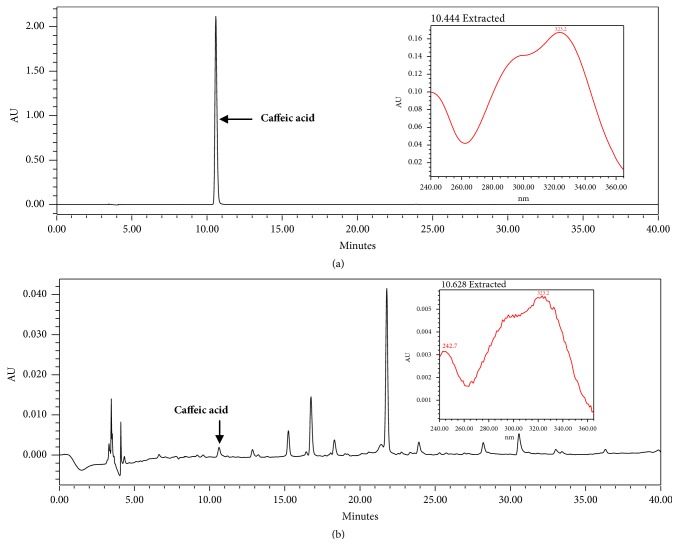
HPLC chromatograms of caffeic acid (a) and wLL (b) detected at 254 nm.

Caffeic acid was used as a standard marker of wLL. As shown Figure 1, the retention time of the caffeic acid was 10.62 min (Figure 1(a)). The chromatographic peak of the wLL was 10.62 min in a wave- length of 254 nm (Figure 1(b)).

### 3.2. wLL Inhibits the RANKL-Induced Osteoclastogenesis in RAW 264.7 Cells

**Figure 2:**
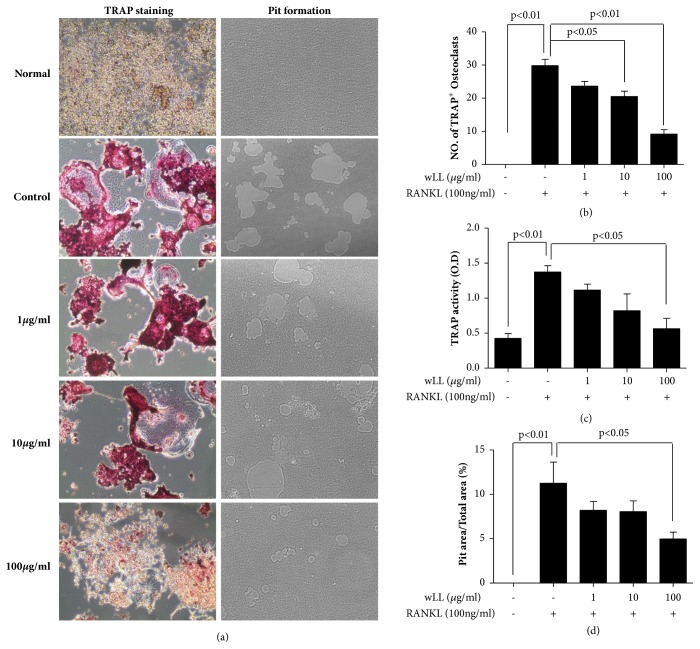
**wLL inhibits RANKL-induced osteoclast differentiation and bone resorption in RAW 264.7 cells**. RAW 264.7 cells were seeded at 5 × 10^3^/well on a 96-well plate (for osteoclast differentiation) or osteo assay surface plate (for bone resorption). After 24 hours, RAW 264.7 cells were incubated with RANKL (100ng/ml) for 4 days with or without the presence of LL (1, 10, and 100 *μ*g/mL). For osteoclast staining, cells were fixed with 4% formalin and stained using TRAP kit. To measure the bone resorption activity, the RAW 264.7 cells on osteo assay plate were removed with 4% sodium hypochlorite. Pit area was subjected to visualization with inverted microscope. ((a) left) TRAP-positive cells detected by TRAP staining, and ((a) right) pit area on the osteo assay plate observed under the inverted microscope (original magnification ×100). (b) TRAP-positive cells with more than three nuclei, and (c) TRAP activity of media measured by an ELISA reader. (d) Pit areas measured using Image J software. Data represent the mean ± SEM of the three independent experiments.

To choose the optimal experimental conditions, the RAW 264.7 cells were cultured with various concentrations of wLL (1, 10 and 100 *μ*g/mL) for 24 hours. As a result, wLL was not toxic to the RAW 264.7 (Supplementary Figure 1). Therefore, we investigated the effect of wLL in nontoxic concentrations for RANKL-induced osteoclast differentiation. To investigate the inhibitory effect of wLL for osteoclastogenesis, cultured cells were stained for TRAP. As compared with normal, control was significantly increased the formation of TRAP-positive osteoclasts, and wLL inhibited TRAP activity as well as the formation of TRAP-positive osteoclasts (Figures 2(a), 2(b), and 2(c)). To evaluate the effect of wLL on bone resorption function by osteoclast, resorptive pit areas were measured. As a result, pit area of control was increased compared to normal. wLL inhibited the pit formation by osteoclasts (Figures 2(a) and 2(d)).

### 3.3. wLL Inhibits the Expression of TRAF-6 in RANKL-Induced RAW 264.7 Cells

**Figure 3:**
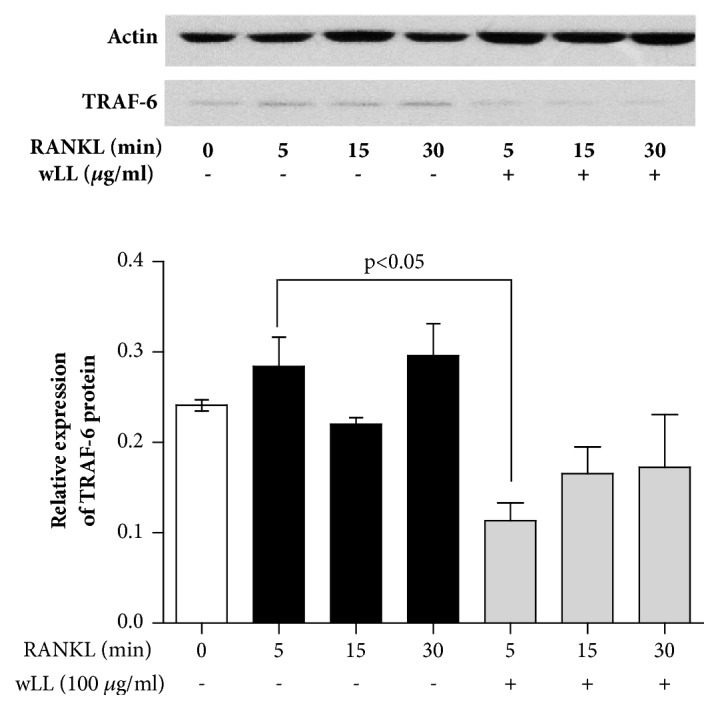
**wLL inhibits the expression of TRAF-6 in RANKL-induced RAW 264.7 cells**. RAW 264.7 cells were seeded at 2 × 10^6^/well on a 60 mm culture plate. After 24 hours, RAW 264.7 cells were incubated with RANKL (100 ng/ml) for indicated times with or without the presence of LL (100 *μ*g/mL). The cells were lysed with RIPA buffer, and expression of TRAF-6 was evaluated with western blot. (A) Expression of TRAF-6. (B) Expression of TRAF-6 normalized to actin. Data represent the mean ± SEM of the three independent experiments.

The combination of RANKL and RANK recruits TRAF 6 and induced the expression of the c-fos and NFATc-1, which are necessary for osteoclast formation [17]. To investigate whether wLL regulate the TRAF-6 recruit, the protein level of TRAF-6 was analyzed with western blot. As shown in Figure 3 TRAF-6 tented to increase at 5min after RANKL treatment. wLL (100ug/ml) markedly declined the expression of TRAF-6 in 5 min (Figure 3).

### 3.4. wLL Inhibits the Expression of NFATc1 and c-fos in RANKL-Induced RAW 264.7 Cells

**Figure 4:**
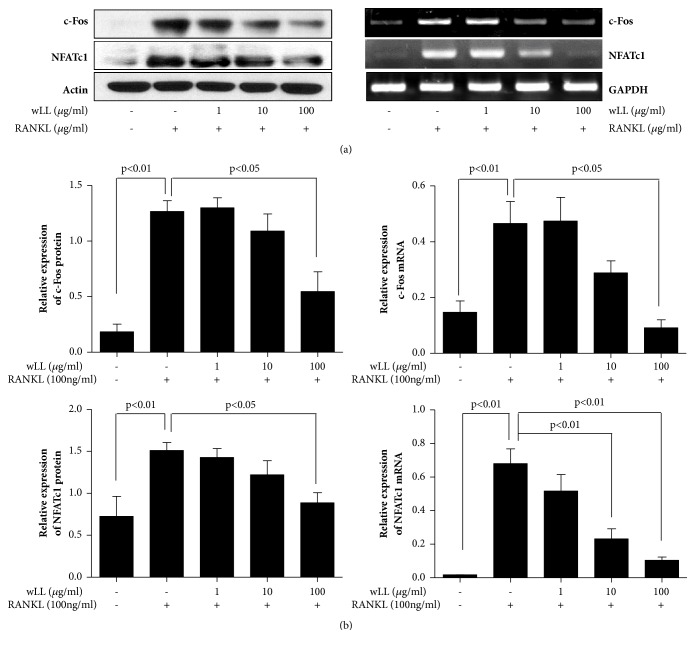
**wLL inhibits the expression of c-fos and NFATc-1 in RANKL-induced RAW 264.7 cells**. RAW 264.7 cells were seeded at 5 × 10^5^/well on a 60 mm culture plate. After 24 hours, RAW 264.7 cells were incubated with RANKL (100 ng/ml) for 4 days with or without the presence of LL (1, 10, and 100 *μ*g/mL). The cells were lysed with RIPA buffer; protein expressions of c-fos and NFATC-1 were evaluated with western blot. To analysis of mRNA expression, the cells were extracted using a TriZol kit, and mRNA expressions of c-fos and NFATc1 were determined by RT-PCR. (a) Protein and mRNA expression of c-fos and NFATc-1. (b) Protein expression of c-fos and NFATc1 normalized to actin; mRNA expression of c-fos and NFATc1 normalized to GAPDH. Data represent the mean ± SEM of the three independent experiments.

To investigate whether wLL regulates the activity of transcription factors such as c-fos and NFATc1 in RANKL-induced osteoclastogenesis, the protein and mRNA expression of c-fos and NFATc1 were detected using Western blot and PCR, respectively. wLL inhibited the expressions of protein as well as the mRNA of c-fos and NFTAc1 (Figure 4).

### 3.5. wLL Inhibits the Expression of Osteoclast-Related Genes in RANKL-Induced RAW 264.7 Cells

**Figure 5:**
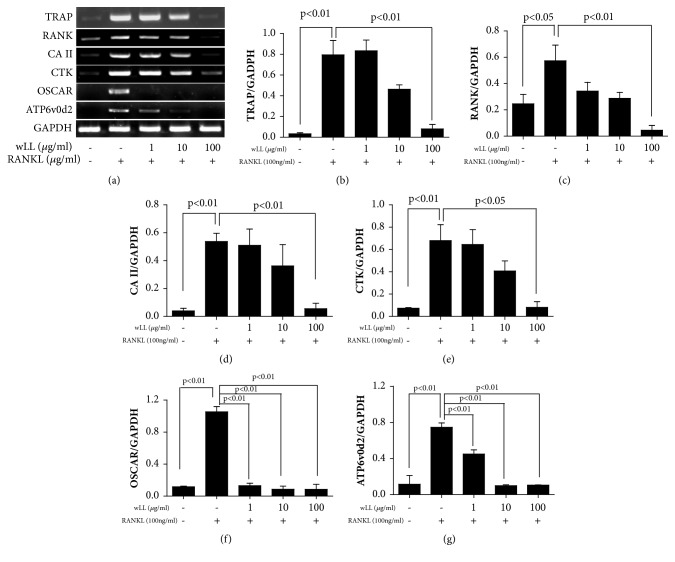
**wLL inhibits expression of osteoclast-related genes of RANKL-induced RAW 264.7 cells**. RAW 264.7 cells were seeded at 5 × 10^5^/well on a 60 mm culture plate. After 24 hours, RAW 264.7 cells were incubated with RANKL (100 ng/ml) for 4 days with or without the presence of LL (1, 10, and 100 *μ*g/mL). The cells were extracted using a TriZol kit, and mRNA expression of osteoclast-related genes was determined by RT-PCR. (a) mRNA expressions of osteoclast-related genes. (b) mRNA expressions normalized to GAPDH. Data represent the mean ± SEM of the three independent experiments.

We also measured the expression of osteoclast-related genes to evaluate the inhibitory effect of wLL. As shown in Figure 5, RANKL significantly increased the expression of osteoclast-related genes. On the other hand, wLL inhibited the expression of osteoclast-related genes, such as TRAP, RANK, CA II, CTK, and OSCAR, involved with bone resorption, and of ATP6v0d2 related to osteoclast fusion.

### 3.6. wLL Suppresses OVX-Induced Bone Loss

**Figure 6:**
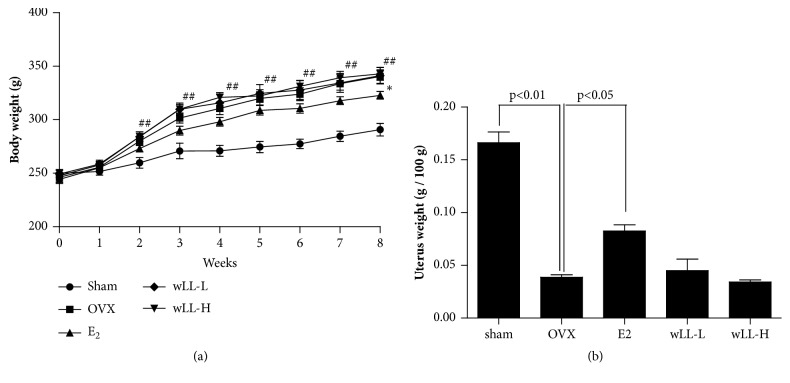
**Effect of wLL on OVX-induced osteoporosis rats**. (a) Body weight was measured once a week for 8 weeks in sham group (circle), OVX group (square), E2 group (triangle): administration of 17*β*-estradiol at 100 *μ*g/kg after OVX, wLL-L group (diamond): administration of wLL at 15.2 mg/kg after OVX, and wLL-H group (inverted triangle): administration of wLL at 152 mg/kg after OVX. (b) Uterus weight measured after sacrificing the rats. Data represent the mean ± SEM of eight independent experiments.

**Figure 7:**
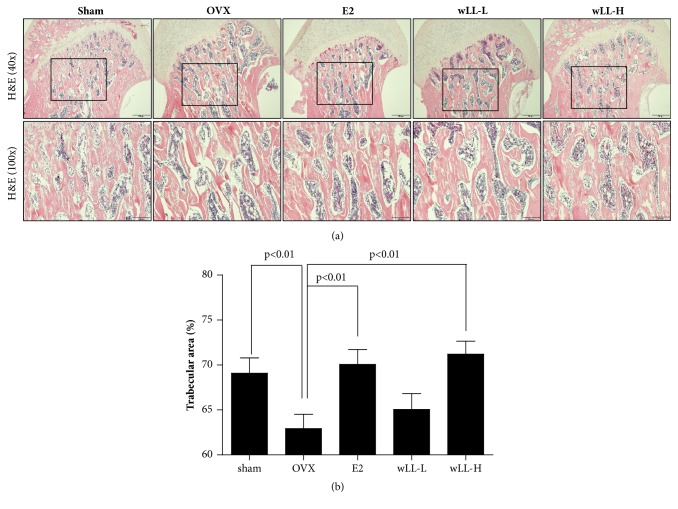
**wLL inhibits bone loss of trabecular area in OVX-induced osteoporosis rats**. (a) Representative histological image of femoral head from OVX-induced osteoporosis rat using H&E staining. Sham group: sham operated group, OVX group: ovariectomized group, E2 group: administration of 17*β*-estradiol at 100 *μ*g/kg after OVX, wLL-L group: administration of wLL at 15.2 mg/kg after OVX, and wLL-H group: administration of wLL at 152 mg/kg after OVX. Scale bars are 500 *μ*m (40x) and 200 *μ*m (100x). (b) Trabecular area measured using Image J software. Data represent the mean ± SEM of eight independent experiments.

The OVX model is the most frequently used animal model for the study of menopausal bone loss. We used an OVX rat model in this study to investigate the effect of wLL for osteoporosis. It has been found that a deficiency of E2 by OVX causes weight gain and uterine atrophy. As expected, after 8 weeks of OVX, the body weight of the OVX group significantly increased compared with the sham group. In the E2 group, the increase in body weight was inhibited, and there was no impact on the wLL-L and wLL-H groups (Figure 6(a)). Also, uterine contraction was observed in all groups except the E2 group (Figure 6(b)). To determine whether wLL affects the bone loss, we measured the trabecular area of femur using a hematoxylin and eosin stain. Trabecular areas of the femur in OVX group decreased in comparison with the sham group. The E2 and wLL-H groups significantly increased compared to the OVX group, but the wLL-L group was not effected (Figure 7).

## 4. Discussion

LL is a perennial herb that belongs to the Lamiaceae family and has traditionally been used for medicinal purposes. Although LL is used for gynecological conditions, there are few studies on the pharmacological action and mechanism of LL [11]. LL contains glycoside; saponins; lycopose; phenol; and flavonoids such as chrysoeriol, luteolin, and quercetin, and caffeic acid [18, 19]. Several studies have reported that flavonoids such as luteolin, quercetin, and kaempferol inhibit osteoclast differentiation and bone loss in OVX-induced osteoporosis [20–22]. Therefore, we have hypothesized that flavonoid-rich LL is associated with a reduced risk of osteoporosis, which is a condition related to gynecological diseases.

RANKL is one of the most important cytokines for osteoclastogenesis; it binds to RANK expressed in osteoclast progenitor cells and leads to osteoclast differentiation [7]. Murine macrophage RAW 264.7 cells are differentiated into multinucleated TRAP-positive cells via RANKL treatment, and these cells cause bone resorption. In this study, RAW 264.7 cells were differentiated into TRAP-positive osteoclasts by RANKL, and pit formations—which were used to measure capability of bone resorption by these activated cells—increased. However, wLL suppressed the differentiation of RAW 264.7 into TRAP-positive cells and inhibited the TRAP secretion into the culture media, thereby decreasing the pit formation.

To investigate the mechanism for effect of wLL, we analyzed the expression of TRAF-6, transcription factors, osteoclast-related genes such as TRAP, CA II, CTK, OSCAR, and ATP6v0d2. RANKL-RANK interaction recruits TRAF 6, and it leads to activation of MAPK pathway related to TRAF-6 mediated-osteoclastogenesis. ERK, JNK, and p38 subfamilies of MAPKs, related to the osteoclast differentiation and bone resorption. Phosphorylation of ERK can induce the activation and expression of the c-fos [23], JNK and p38 are related only osteoclastogenesis [24]. In the present study, wLL inhibited the activation of TRAF-6 but did not show effect in MAPKs signaling pathway. Therefore, we investigate the c-fos and NFATc1 signal pathway. Also the RANKL-RANK interaction is involved in activation of c-fos expression in osteoclast precursor [25]. c-fos is critical for transcriptional activation of NFATc-1, activated c-fos leads to activation of NFATc-1, a key regulators that induced osteoclast differentiation, fusion, and activation [6, 17, 26]. c-fos or NFATc-1 deficient mice exhibit osteopetrosis due to deficiency of osteoclast differentiation [27, 28]. Thus, c-fos and NFATc-1 play critical roles in osteoclastogenesis. In this study, RANKL increased the expression of c-fos and NFATc-1 in mRNA and the protein level, and wLL significantly decreased expression of NFATc-1 and c-fos. These results indicate that the wLL inhibits RANKL-induced osteoclastogenesis by blocking of c-fos and NFATc-1 pathway. In addition, NFATc-1 can translocate from the cytoplasm to the nuclear, leading to the target genes related to osteoclast differentiation and activation, such as ATP6v0d2, CA II, CTK, TRAP, and OSCAR [29, 30]; CA II is a hormone expressed at the early stages of osteoclastogenesis and plays an important role in cellular pH regulation. CA II acidifies the resorption lacuna for demineralization of bone [31]. During osteoclastogenesis, CTK and TRAP are secreted into the resorption lacuna, acidified sealed area. CTK is an enzyme that digests type I collagen, which is a major component of the bone matrix [32], and its overexpression causes osteoporosis by inducing excessive bone loss [33]. TRAP is a bone resorption marker that is expressed by activated osteoclasts. In mice, a TRAP deficiency has been found to cause osteopetrosis, with increased bone mineral density [32]. Cell fusion-mediated multinucleated cell formation is a necessary process for osteoclast maturation. Atp6v0d2 plays an important role in osteoclast as well as macrophage cell-cell fusion [34]. In this study, we analyzed the expression of osteoclast-related genes using RT-PCR. We found that wLL reduced the osteoclastogenesis-related genes like CA II, TRAP, RANK, CTK, OSCAR, and Atp6v0d2. These results mean that wLL inhibit the differentiation, activation, and bone resorption of mature osteoclasts as well as the formation of multinucleated osteoclasts by cell-cell fusion via block of c-fos/NFATc-1 pathway in RANKL-induced osteoclastogenesis.

Osteoporosis in postmenopausal woman is caused by loss of bone mass due to persistent estrogen deficiency [35]. OVX-induced osteoporosis models are widely used for the study of postmenopausal osteoporosis [36], and in this study we measured trabecular area, uterus weight, and body weight after oral administration of wLL for 8 weeks in an OVX-induced osteoporosis rat. We found that wLL did not affect body weight, uterus weight, and estrogen concentration compared with the OVX group. In addition, wLL suppressed loss of the trabecular area in the OVX femur. These results show that wLL may prevent postmenopausal osteoporosis caused by an estrogen deficiency.

## 5. Conclusion

wLL suppressed osteoclastogenesis and bone resorption by RANKL in a concentration-dependent manner by modulating the NFATc/c-fos pathway in vitro, and wLL inhibited bone loss in the OVX-induced osteoporosis model in vivo. In conclusion, wLL was able to protect bone loss in the OVX-induced osteoporosis model without the influence of hormones like estrogen. Therefore, wLL may be a reasonable alternative for treating postmenopausal osteoporosis.

## Figures and Tables

**Table 1:** Primers, sequences, and PCR conditions.

Primer name	Primer Sequence	Annealing Tm (°C)	cycle
*TRAP*	5′-ACTTCCCCAGCCCTTACTACCG-3′ 5′-TCAGCACATAGCCCACACCG-3′	58°C	32
*NFATc1*	5′-TGCTCCTCCTCCTGCTGCTC-3′ 5′-CGTCTTCCACCTCCACGTCG-3′	58°C	32
*c-Fos*	5′-ATG GGC TCT CCT GTC AAC AC-3′ 5′-GGCTGCCAA AATAAACTCCA-3′	58°C	33
*RANK*	5′-AAACCTTGGACCAACTGCAC-3′ 5′-ACCATCTTCTCCTCCCGAGT-3′	53°C	32
CAII	5′-CTCTCAGGACAATGCAGTGCTGA-3′ 5′-ATCCAGGTCACACATTCCAGCA-‘3	58°C	33
CTK	5′-AGGCGGCTATATGACCACTG-3′ 5′-CCGAGCCAAGAGAGCATATC-‘3	58°C	28
OSCAR	5′-CTGCTGGTAACGGATCAGCTCC CCAGA-3′ 5′-CCAAGGAGCCAGAACCTTCGAAACT-3′	53°C	40
*ATP6v0d2*	5′-ATGGGGCCTTGCAAAAGAAATCTG-3′ 5′-CGACAGCGTCAAACAAAGGCTTGTA-3	58°C	30
*GAPDH*	5′-ACTTTGTCAAGCTCATTTCC-3′ 5′-TGCAGCGAACTTTATTGATG-3′	57°C	30

## Data Availability

The data used to support the findings of this study are available from the corresponding author upon request.

## Abbreviations

*α*-MEM: Medium essential medium *α*
CTK: Cathepsin K
DMEM: Dulbecco's modified eagle's medium
E2: 17*β*-estradiol
FBS: Fetal bovine serum
NFATc1: Nuclear factor of activated T-cell cytoplasmic 1
OVX: Ovariectomized
PCR: Polymerase chain reaction
RANKL: Receptor activator of the nuclear factor-kappa B ligand
TRAP: Tartrate-resistant acid phosphatase
TRAF6: Tumor necrosis factor receptor-associated factor 6
CA II: Carbonic anhydrase II
LL: * Lycopus lucidus* Turcz
wLL: Water extract of* Lycopus lucidus* Turcz
HPLC: High performance liquid chromatography
cDNA: Complementary DNA
ATP6v0d2: d2 isoform of vacuolar ATPase Vo domain
OSCAR: Osteoclast-associated receptor.

## Supplementary Materials

Supplementary Materials Supplementary Figure 1: wLL was not toxic to the RAW 264.7. Cell viability was analyzed with MTS assay. Data represent the mean ± SEM of the three independent experiments.
