# Supplementary material for: Lycopus lucidus Turcz Inhibits the Osteoclastogenesis in RAW 264.7 Cells and Bone Loss in Ovariectomized Rat Model
Source: Evid Based Complement Alternat Med. 2019 Feb 21;2019:3231784. doi: 10.1155/2019/3231784 (PMC6409043; doi:10.1155/2019/3231784)
Supplement: Supplementary Materials — Supplementary Figure 1: wLL was not toxic to the RAW 264.7. Cell viability was analyzed with MTS assay. Data represent the mean ± SEM of the three independent experiments. [file 3231784.f1.docx]

**Supplementary Information**

***Lycopus lucidus* Turcz inhibits the osteoclastogenesis in RAW 264.7 cells and bone loss in ovariectomized rat model**

Da-Won Jeong^1, #^, Eun-Young Kim^1, #^, Jae-Hyun Kim^1^, Bina Lee^1^, Jae Ho Park^2^, Hyuk-Sang Jung^1^, Youngjoo Sohn^1,^ *

**Supplementary figure 1. wLL were treated with various concentration in RAW 264.7 cells**

RAW 264.7 cells were seeded in 96 well plate and treated with wLL (1, 10 and 100 μg/mL) for 24h. Then, MTS reagent was added to the plate. After 2h, Optical density at 490nm. The viability of untreated cell was regarded as 100%.
